# What Is the Minimum Effective Volume of Local Anaesthetic Applied in Brachial Plexus Blockage With an Axillary Approach Under Ultrasonography Guidance?

**DOI:** 10.7759/cureus.16865

**Published:** 2021-08-03

**Authors:** Necati A Erdogmus, Semih Baskan, Musa Zengin, Gokhan Demirelli

**Affiliations:** 1 Intensive Care Clinic, Ankara University Faculty of Medicine, Ankara, TUR; 2 Anesthesiology and Reanimation Clinic, Ankara City Hospital, Ankara, TUR; 3 Anesthesiology and Reanimation, University of Health Sciences, Ankara Atatürk Chest Diseases and Thoracic Surgery Training and Research Hospital, Ankara, TUR; 4 Anesthesiology and Reanimation Clinic, Bafra State Hospital, Samsun, TUR

**Keywords:** axial approach, brachial plexus blockage, minimum effective anesthetic volume, regional anesthesiology, ultrasonography (usg)

## Abstract

Peripheral nerve blocks with the use of ultrasonography (USG) allow visualisation of both the structures and nerves and make the block administrations safe, quick, and comfortable. However, few publications concerning the minimum local anesthetic (LA) volume are capable of providing blocks. This study aimed to find the minimum effective LA volume in brachial plexus blockage administrations with an axillary approach accompanied by ultrasonography in hand, elbow, and forehand operations.

Materials and Method

The study included a total of 55 patients (classified as American Society of Anesthesiologists (ASA) I-II) who underwent hand surgery by administering USG-guided axillary brachial plexus blockage. The ulnar, median, and radial nerves were located, and the minimum effective LA volume was investigated starting with a total of 21 ml of bupivacaine 0.5%. After accomplishing the blockage, the volume was decreased by 0.5 ml for each nerve. Block administration time, block onset times, anesthesia times, and time to first analgesic requirement were recorded.

Results

The minimum effective LA volume for each nerve was 2.5 ml for a total of 7.5 ml. In comparing block administration times, there were no differences between high or low volume groups. It was found that sensory block onset time was 17 minutes for 7.5 ml and 11 minutes for 21 ml; sensory block regression time was six hours for 7.5 ml and 10.4 hours for 21 ml, respectively. This regression was statistically significant. The first analgesic requirement was 5.8 - 16.6 hours, respectively, for each group.

Conclusion

In the administration of an USG-guided axillary block, sufficient anesthesia can be achieved by administering 2.5 ml of bupivacaine 0.5% for each nerve. However, it might be kept in mind that motor and sensory block onset time will be extended and regression time and time to the first analgesic requirement will be shorter with this volume. In addition, more advanced studies must be done for the determination of the optimum volume which can be used.

## Introduction

Regional anaesthesia is currently accepted as a preferred application to general anaesthesia in suitable patients because of the provision of analgesia perioperatively and postoperatively, as well as reductions in perioperative morbidity, postoperative length of hospital stay, and costs [1].

With low rates of side effects, peripheral nerve blocks have recently started to be increasingly widely used in the provision of surgical anaesthesia levels and postoperative analgesia.

The plexus nerves can be blocked from the several desired points along the pathway. A block from various levels of the brachial plexus is sufficient to provide anaesthesia of all the deep structures of the upper extremity and all the skin from above the distal arm as far as the mid-arm. The patient’s wishes, together with the knowledge and experience of the surgeon and anesthesiologist, play an important role in the application of this method [1].

Over the years, several techniques have been used for the implementation of a successful and reliable block. Recently, blocks applied under ultrasonography (USG) guidance have become widespread [2]. Ultrasound at a high frequency offers clearer visualisation of surface tissues and has the significant advantages of reducing the local anesthetic (LA) dose, the risk of LA toxicity, and associated complications in the application of regional block [3-4].

Brachial plexus block with an axillary approach (ABPB) blocks the radial, ulnar, and median nerves at the same time in forearm, wrist, and hand surgery. In plexus blocks applied with conventional methods, a LA of 30 - 40 ml volume is used. In comparison with conventional methods, it has been reported that with the use of USG, block success is increased, the time to onset of anaesthesia for surgery is shortened, and the block can be applied with a lower volume of LA. However, there is no consensus as to the lowest volume of LA that is effective.

This study aimed to investigate the lowest volume of LA that is effective in ABPB applied under USG guidance in surgical interventions to the forearm, wrist, and hand regions of the upper extremity.

## Materials and methods

Approval for the study was granted by the Ethics Committee of Ankara Numune Training and Research Hospital (ID: E14-337). The study included 55 patients classified as American Society of Anesthesiologists (ASA) I-II, aged 18 - 65 years, who were scheduled to undergo hand, wrist, or forearm surgery. Informed consent was obtained from all the study participants. All the researchers participating in this prospective, controlled, single-centre study signed the Helsinki Declaration. Exclusion criteria were patients with coagulopathy, pregnancy, LA allergy, neurological or neuromuscular disease, infection or wound scar in the application site, impaired mental status, ASA III-V, body mass index (BMI) > 35 kg/m2, or non-acceptance of the procedure.

For all patients, age, gender, height, weight, and ASA score were recorded. On admission to the operating room, standard monitorisation was applied (electrocardiogram, pulse oximetry, and non-invasive blood pressure). Before the block, intravenous (IV) premedication was administered (0.1 mg/kg midazolam and 1 µg/kg fentanyl).

All the blocks were applied by the same practitioner who was experienced in the application of blocks under USG guidance. The USG application was made with a LOGIQ e machine (General Electric Healthcare Systems, Waukesha, WI, USA) with a wideband and multifrequency linear probe (HFL 38, 13-6 MHz). The patient was positioned supine and the arm was placed in 90° abduction. After antiseptic cleaning of the probe and the application area, the block was applied with a Locoplex 50 mm stimulator needle (Vygon SA, Ecouen, France). LA was administered via bupivacaine solution 0.5%.

With the linear probe in the transverse plane, the best view was sought of the brachial plexus from the lateral edge of the pectoralis major muscle. After localising the axillary artery, the terminal branches of the brachial plexus with the surface course, the median nerve (superficial and lateral of the artery), the ulnar nerve (superficial and medial of the artery), and the radial nerve (posterior lateral or medial of the artery) were located. By entering the needle from the side of the probe, visualisation of the application was provided along the long axis. The radial nerve was selected as the first target as it was in the posterior of the artery. The median and ulnar nerves were then located and local anaesthetic was applied. The musculocutaneous nerve was also blocked using 2 ml of 0.5% bupivacaine, the standard in all patients.

LA application was made starting from the ceiling volume (21 ml administered as 7 ml per each nerve). For each volume of LA, five patients were to be included in the study. Thus, the block needed to be successful in at least three of the five patients for that volume to be able to be accepted as a successful volume to be used for the block. In the first five patients, a total of 21 ml LA was applied as 7 ml to each nerve. After the determination of successful blocks at that volume, the total LA volume administered to the subsequent five patients was reduced by 1.5 ml (a reduction of 0.5 ml for each nerve).

The researcher testing the block was blinded to the study protocol. The success of the block was measured according to a 3-point scale test using sensory and motor block measurements [5]. By comparison, with the contralateral arm, scoring was made as 0 = no block, 1 = analgesia (touch sensation present, heat sensation absent), and 2 = full sensory block (no touch sensation). For the evaluation of motor block, a 3-point scale was used with values of 0 = no block, 1 = partial motor block, and 2 = full motor block. The evaluation was made respectively of the radial nerve with the loss of thumb abduction movement, the median nerve with thumb adduction, and the ulnar nerve with thumb opposition.

Evaluations were made every five minutes (mins) in the first 60 mins. Patients determined to have an unsuccessful block within 60 mins were administered general anaesthesia and were withdrawn from the study evaluation.

Measurement of the block application time was started from the time the block needle touched the skin and finished after completion of the local anaesthetic injection. The duration of anaesthesia and the duration of surgery were measured. The motor and sensory block onset to recovery times were accepted as the moment the score per nerve receded from 2 to 1 according to the 3-point scale and cold test and the block recovery times as the time when the score per nerve was 0 (zero). Patient postoperative pain was evaluated with a visual analog scale (VAS) from 0 (no pain) to 10 (intolerable pain) on a ruler. The time of requirement for the first analgesia was accepted as a VAS score of > 4. Operating time was recorded, and patient and surgeon satisfaction were evaluated as very good, good, moderate, and poor.

Statistical analysis

In the calculation of the sample size, the Dixon and Massey method was used. According to this calculation, when n = 2(SD/SEM)²; n: sample size, SD: standard deviation, SEM: standard error of the mean, (SD = 5 ml, SEM = 1.0 ml), the number of patients was found to be 50. The effect size was calculated with G * Power 3.0.10 statistics software (Heinrich Heine University, Düsseldorf, Germany), and 50 patients were found to be sufficient with an effect size = 0.4, α = 0.05, degrees of freedom (df) = 48, and power 0.85 (power (1-β)) [6]. To allow for 10% error in the application, a total of 55 patients were included in the study.

Statistical analyses of the study data were applied with the IBM Statistical Package for Social Sciences (SPSS), v. 21.0 software (IBM SPSS Statistics for Windows, Armonk, NY). Descriptive statistical methods were used when evaluating the study data (frequency, percentage, mean, standard deviation). Conformity of the data to normal distribution was evaluated with the Kolmogorov-Smirnov test and data were found to be of normal distribution. In the comparisons between volumes, a one-way analysis of variance (ANOVA) was used. To determine from which volume group a difference originated, post hoc tests (Least Significant Difference (LSD) and Tukey's Honestly Significant Difference (HSD)) were applied. Relationships between volume and times were examined with the Pearson correlation analysis. A value of p < 0.05 was accepted as statistically significant.

## Results

The demographic characteristics, ASA scores of the patients included in the study, the duration of anaesthesia and surgery, the starting and finishing times of the blocks, and the time of requirement for the first analgesia are shown in Table 1.

**Table 1:** Characteristics of the Study Participants and Duration of Operation ASA: American Society of Anesthesiologists; BMI: body mass index; cm: centimetre; kg: kilogram; hr: hour; mins: minutes; SD: standard deviation; secs: seconds

	Mean ± SD
Female (n)	17
Male (n)	38
Age (years)	42.1 ± 16.4
Weight (kg)	73.5 ± 11.8
Height (cm)	170.2 ± 8.7
BMI	25.3 ± 3.7
ASA I (n)	21
ASA II (n)	34
Duration of operation (mins)	71.1 ± 36.7
Duration of block application (secs)	154.5 ± 56.8
Duration of anaesthesia (mins)	76.4 ± 37.1
Onset of sensory block (mins)	11.4 ± 6.9
Onset of motor block (mins)	12.0 ± 7.0
Time to recovery of sensory block (hr)	8.2 ± 2.1
Time to recovery of motor block (hr)	8.4 ± 2.6
Time to requirement for first analgesia (hr)	12.8 ± 4.3

**Figure 1:**
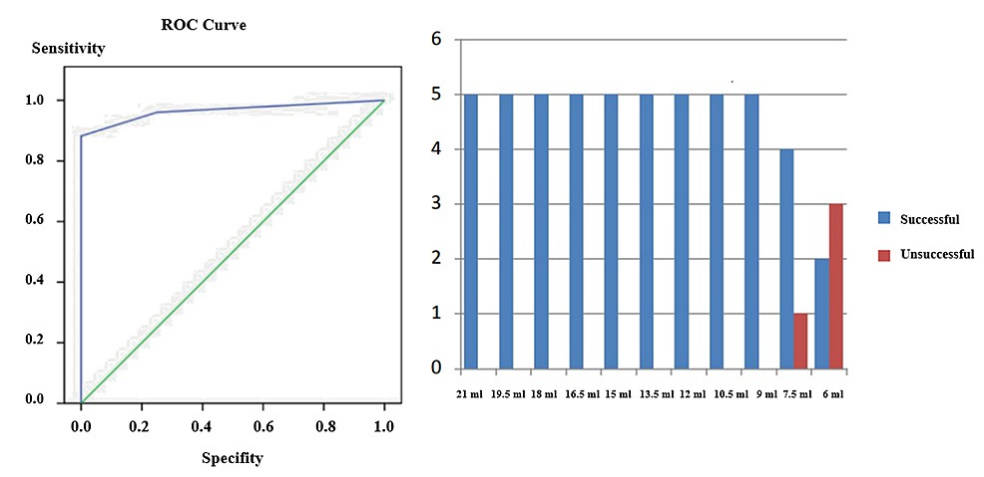
ROC analysis and the cutoff point of the volume (dose) value for a successful block Evaluation with ROC analysis of the volume and block success. Minimum effective volume. As a result of the evaluation made with ROC analysis, the cutoff point of the volume (dose) value for a successful block was found to be ≥ 7.5 ml (AUC = 0.966, p < 0.001, 95% CI: 0.878 - 0.996, cutoff = volume ≥ 7.5 ml). AUC: area under the curve; CI: confidence interval; ROC: receiver operating characteristic

As a result of the evaluation made with receiver operating characteristic (ROC) analysis, the cutoff point of the volume (dose) value for a successful block was found to be ≥ 7.5 ml (area under the curve (AUC) = 0.966, p < 0.001, 95% confidence interval (CI): 0.878 - 0.996, cutoff = volume ≥ 7.5 ml) (Figure 1).

No statistically significant difference was determined in the block application duration or the anaesthesia duration according to the volume applied (p > 0.05), while statistically significant differences were determined in the periods of time measured (p < 0.05) (Table 2). Multiple comparison tests (post hoc) were applied to determine these differences.

**Table 2:** Comparison of Times According to Volume (Mean ± SD) hrs: hours; mins: minutes; SD: standard deviation; secs: seconds

Volume	Duration of block application (secs)	Duration of anaesthesia (mins)	Onset of sensory block (mins)	Onset of motor block (mins)	Time to recovery of sensory block (hrs)	Time to recovery of motor block (hrs)	Time of requirement for first analgesia (hrs)
6.0 ml	191.0 ± 43.8	59.0 ± 11.4	25.0 ± 9.4	26.0 ± 9.6	4.8 ± 1.3	4.6 ± 0.9	5.8 ± 1.3
7.5 ml	181.0 ± 87.9	86.0 ± 42.9	17.0 ± 10.4	17.0 ± 10.4	6.0 ± 1.9	6.2 ± 1.9	8.6 ± 3.0
9.0 ml	156.0 ± 37.3	85.0 ± 57.6	11.0 ± 2.2	11.0 ± 2.2	7.8 ± 1.5	8.4 ± 2.7	12.8 ± 2.4
10.5 ml	130.0 ± 35.5	81.0 ± 36.0	10.0 ± 3.5	10.0 ± 3.5	8.6 ± 1.9	8.8 ± 1.8	14.6 ± 4.0
12.0 ml	139.4 ± 47.4	98.0 ± 19.6	10.0 ± 6.1	11.0 ± 6.5	7.8 ± 2.5	8.6 ± 3.0	11.6 ± 3.5
13.5 ml	159.0 ± 82.4	50.0 ± 14.6	7.0 ± 2.7	10.0 ± 3.5	8.2 ± 0.4	7.4 ± 0.9	11.6 ± 1.1
15.0 ml	105.0 ± 19.0	70.0 ± 30.2	8.0 ± 2.7	9.0 ± 2.2	9.0 ± 2.0	8.8 ± 2.2	12.6 ± 1.5
16.5 ml	136.0 ± 51.2	121.0 ± 15.6	10.0 ± 0.0	11.0 ± 2.2	9.4 ± 1.7	9.8 ± 1.3	14.6 ± 5.4
18.0 ml	183.2 ± 65.9	61.0 ± 38.8	8.0 ± 2.7	8.0 ± 2.7	9.6 ± 1.1	9.0 ± 1.0	15.4 ± 1.1
19.5 ml	140.0 ± 38.4	56.0 ± 30.7	8.0 ± 4.5	8.0 ± 4.5	8.8 ± 1.6	9.0 ± 1.4	16.2 ± 2.5
21.0 ml	179.0 ± 65.8	73.0 ± 49.2	11.0 ± 2.2	11.0 ± 2.2	10.4 ± 1.1	12.0 ± 3.5	16.6 ± 5.2
P-value	0.375	0.114	0.004	0.016	0.003	0.002	0.001

**Figure 2:**
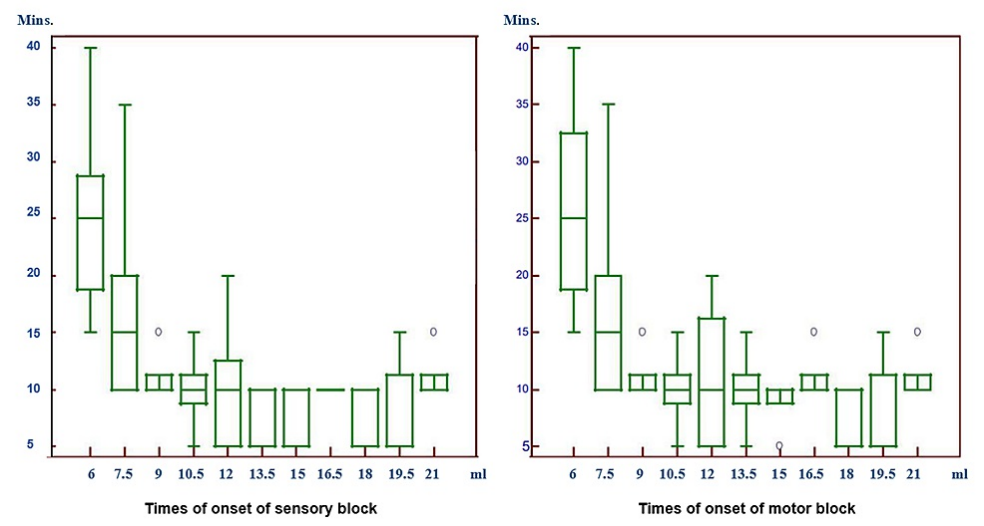
Times of the onset of sensory and motor blocks In the comparison of the times of the onset of a sensory block, this period was determined as the longest in the patients applied with the lowest volume of 6.0 ml of local anesthetic (LA), and this difference was statistically significant. In the patients administered with a greater volume, the rate of the decrease in these periods was found to be statistically significant. In the comparison of the times of the onset of a motor block, the difference between the patients administered with 6.0 ml LA and 9.0 ml or higher volumes of LA, and the difference between patients administered with 7.5 ml LA and those administered with 15.0 ml, 18.0 ml, and 19.5 ml LA were found to be statistically significant. The time to the onset of a motor block of patients administered with 6.0 ml LA and 7.5 ml LA was found to be longer compared to the others.

In the comparison of the times of the onset of a sensory block, this period was determined as longest in the patients applied with the lowest volume of 6.0 ml LA, and this difference was statistically significant (Figure 2). In the patients administered with a greater volume, the rate of the decrease in these periods was found to be statistically significant. In the comparison of the times of the onset of a motor block, the difference between the patients were found to be statistically significant (Figure 2).

**Figure 3:**
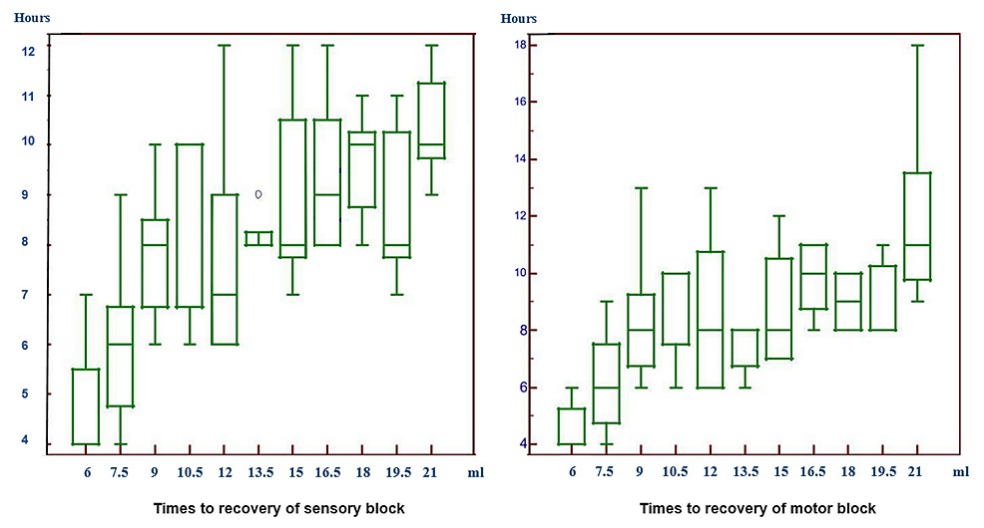
Times to the recovery of the sensory and motor blocks In the comparison of the times to the recovery of a sensory block, a statistically significant difference was found between patients administered with 6.0 ml LA and those with 9.0 ml and higher volumes of LA. A statistically significant difference was found between patients administered with 7.5 ml LA and those with 10.5 ml, 15.0 ml, 16.5 ml, 18.0 ml, 19.5 ml, and 21.0 ml LA. A statistically significant difference was found between patients administered with 9.0 ml and 21.0 ml LA. A statistically significant difference was found between patients administered with 12.0 ml and those with 18.0 ml and 21.0 ml LA. A statistically significant difference was found and between those administered with 13.5 ml and 21.0 ml. The time to the recovery of a sensory block of patients administered with 6.0 ml and 7.5 ml LA was found to be shorter compared to the others. In the comparison of the times to recovery of motor block, a statistically significant difference was found between patients administered with 6.0 ml LA and those with 9.0 ml and higher volumes of LA. A statistically significant difference was found between patients administered with 7.5 ml LA and those with 10.5 ml, 15.0 ml, 16.5 ml, 18.0 ml, 19.5 ml, and 21.0 ml LA. A statistically significant difference was found between patients administered with 9.0 ml and 21.0 ml. A statistically significant difference was found between patients administered with 12.0 ml and 21.0 ml. A statistically significant difference was found between those administered with 13.5 ml and those with 16.5 ml and 21.0 ml. A statistically significant difference was found between those administered with 15.0 ml and 21.0 ml. The time to recovery of motor block of patients administered with 6.0 ml and 7.5 ml LA was found to be shorter compared to the others.

In the comparison of the times to recovery of a sensory and a motor block, a statistically significant difference was found between patients (Figure 3).

**Figure 4:**
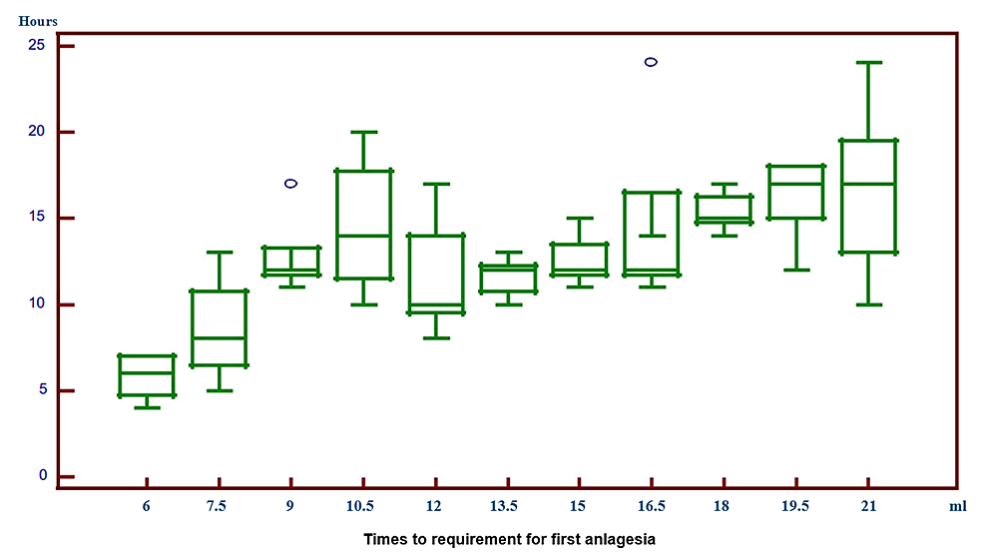
Times to the requirement for the first analgesia In the comparison of the times to the requirement for first analgesia, a statistically significant difference was found between patients administered with 6.0 ml LA and those with 9.0 ml and higher volumes of LA. A statistically significant difference was found between patients administered with 7.5 ml LA and those with 9.0 ml and 19.5 ml LA. A statistically significant difference was found between patients administered with 12.0 ml and 18.0 ml, 19.5 ml, and 21.0 ml. A statistically significant difference was found between those administered with 13.5 ml and those administered with 18.0 ml, 19.5 ml, and 21.0 ml. The requirement for the first additional analgesia of patients administered with 6.0 ml and 7.5 ml LA was found to be earlier compared to the other patients.

In the comparison of the times to requirement for the first analgesia, a statistically significant difference was found between patients (Figure 4).

In the examination of the correlations between the findings and the LA volume applied, statistically significant correlations were found as negative with time to the onset of a sensory block at the level of r = -0.44, negative with time to the onset of a motor block at r = -0.45, positive with time to recovery of sensory block at r = 0.63, positive with time to recovery of a motor block at r = 0.60, and positive with time to a requirement for the first analgesia at r = 0.63 (p < 0.05) (Table 3).

**Table 3:** Pearson Correlation hr: hour; mins: minutes; secs: seconds * p < 0.05,

Pearson Correlation	(1)	(2)	(3)	(4)	(5)	(6)	(7)	(8)
(1) Volume	1.00							
(2) Duration of block application (secs)	-0.07	1.00						
(3) Duration of anaesthesia (mins)	-0.08	0.07	1.00					
(4) Onset of sensory block (mins)	-0.44*	0.13	-0.03	1.00				
(5) Onset of motor block (mins)	-0.45*	0.22	-0.03	0.84*	1.00			
(6) Time to recovery of sensory block (hr)	0.63*	-0.16	0.05	-0.41*	-0.46*	1.00		
(7) Time to recovery of motor block (hr)	0.60*	-0.22	0.10	-0.34*	-0.47*	0.92*	1.00	
(8) Time to requirement for first analgesia (hr)	0.63*	-0.01	0.10	-0.45*	-0.51*	0.68*	0.65*	1.00

Artery punction was observed in only two patients. No other complications were observed. In four patients where the block was accepted as unsuccessful, general anaesthesia was administered with laryngeal mask airway (LMA). No requirement for an additional anaesthesia method was determined in 92.7% of patients. Patient satisfaction was determined as good and very good in 92.7% of patients. Surgeon satisfaction was determined as good and very good at the rate of 92.7% (Table 4).

**Table 4:** Complications, Requirement for an Additional Anaesthesia Method, Patient Satisfaction, and Surgeon Satisfaction

Complications	n (%)
None	53 (96.4%)
Present	2 (3.6%)
Requirement for an Additional Anaesthesia Method	
No	51 (92.7%)
Yes	4 (7.3%)
Patient Satisfaction	
Moderate	4 (7.3%)
Good	24 (43.6%)
Very good	27 (49.1%)
Surgeon Satisfaction	
Moderate	4 (7.3%)
Good	24 (43.6%)
Very good	27 (49.1%)

## Discussion

In peripheral regional anaesthetic techniques, it is customary for high volumes of LA to be used. For example, blockade of the brachial plexus in the axillary region has been described with volumes up to 60 ml [7]. The majority of reported complications are findings of systemic toxicity which could be related to the use of these high volumes. The symptoms can result in severe depression of the central nervous and cardiovascular systems [7]. It is thought that a reduction in the dose and volume of LA used could lead to these unwanted side effects being observed less frequently and that these applications could be used in a more extensive area [7,8]. Modern regional anaesthesia practice should be focused on developing strategies, such as reducing LA volume, to prevent complications [9].

This study aimed to determine the lowest dose of LA which could provide surgical anaesthesia when applied as an axillary brachial plexus nerve block under USG guidance. From the results, the conclusion was reached that a total of 7.5 ml administered as 2.5 ml to each nerve could be a sufficient amount. However, as the amount of LA was decreased, the time to onset of the block was observed to be prolonged, the time to recovery of the block was shortened, and the duration of the block was shortened.

It has been reported that with the use of USG in regional anaesthesia, there was a higher success rate, the time to onset of the block was shorter, and a successful intervention was made with lower doses of LA [10-11]. The use of USG shortens the duration of block application. Chan et al. measured axillary block application time with nerve stimulation as 11.2 ± 4.2 mins, while the time was measured as shorter at 9.3 ± 4.0 mins when the axillary block was applied under USG guidance [10]. In the current study, the block application time was determined as a mean of 154.5 ± 56.8 secs, which was shorter than techniques using a stimulator and consistent with previous findings in the literature [10, 12-13]. There was not determined to be any change in the block application times in the groups applied with low volumes.

Many studies have reported that the volume of LA could be reduced with the use of USG [7, 9, 14-15]. Ferraro et al. applied brachial plexus blockage with an axillary approach under USG guidance in hand surgery and reported that the minimum effective volume which could provide a successful block was 1.56 ml per nerve of bupivacaine 0.5% with 1:200,000 adrenaline [14]. In a study of 19 patients, Harper et al. administered lidocaine 1.5% and 1:200,000 epinephrine to the surroundings of the nerve and reported the mean values to be 2.58 ml - 3.42 ml [15]. However, as the block was not successful in four of the 19 patients at those doses, lower volumes were not applied, and in seven patients, additional blockage was required. Accordingly, in the current study, the number of unsuccessful blocks increased below a volume of a total of 7.5 ml applied as 2.5 ml per nerve. Thus, similar to the previous study, this was determined as the minimum effective volume [15]. It can, therefore, be considered that lower doses may not be clinically reliable.

The current study differed from previous research in that a greater number of patients were included as 55 patients in 11 groups and bupivacaine was selected because it provides a longer blockage duration. Bupivacaine 0.5% was used without the addition of adrenaline and the lowest volume for each nerve was found to be 2.5 ml. However, in another study, Duggan et al. reported that the use of USG in supraclavicular nerve blockage did not reduce the volume of anaesthetic required [16]. Marhofer et al. found the lowest volume to be 4 ml for axillary blockage with mepivacaine 1% [7]. As the experience of the practitioner and the drug concentration to be applied may be effective in the success of the block, similar studies are ongoingly related to the lowest volumes and concentrations [7, 15].

According to Hadzic et al., the use of LA at low volumes could result in intraneural injection [17]. This is associated with difficulties in the measurement of increased nerve diameter. Although this is an unwanted event, block success with low volumes has been reported to have increased the rate of undesired intraneural injection, but this development has not been accepted [18-19]. Consequently, further studies would be useful for the clarification of the area of application of LA. In the application of extraneural block, the two most important conditions are the ability to directly visualise neural structures and the dissemination of the LA. Thus, it can be seen when there has been insufficient distribution of LA and the tip of the needle can be redirected [7].

On the subject of the time to onset of block, O’Donnell et al. applied a 3 ml solution of lidocaine 2% and 1:200,000 adrenaline and reported the mean time to the onset of a block as 5 mins and the mean duration of anaesthesia as 190 mins [9]. Marhofer et al. applied axillary block with a total of 4 ml with mepivacaine 1% and the time to onset of sensory block was reported as 25 mins with the duration of the block as 152 mins [7]. In another study by Harper et al., a mean of 2.58 ml - 3.42 ml per nerve was applied with lidocaine 1.5% and 1:200,000 epinephrine; the time to onset of the block was reported as 22.5 - 26.8 mins and the mean duration of the block as 137 - 183 mins [15]. In the current study, the time to onset of sensory block was 17.0 ± 10.4 mins and the duration of the block was 86.0 ± 42.9 mins with 7.5 ml of bupivacaine 0.5%. In another study, it has been reported that as the LA volume was decreased, so the duration of the block decreased and this shows that blocks applied with low volumes of LA could be selected for day-patient surgical interventions [20]. However, it should be kept in mind that the onset of a block is prolonged in these applications as it could be a disadvantage. Future studies could be planned with low volumes that do not prolong the onset of block compared to higher volumes.

In the reduction of a volume that will be able to provide a block, visualization and location of the radial nerve are difficult and seems to be a restriction of the blockage. It has been proposed that differentiation of the site of the radial nerve with a nerve stimulator could be useful in decreasing the LA volume to be administered [20]. In the current study, it was determined that the use of USG guidance alone could be useful in this issue compared to conventional methods. In patients determined to have an unsuccessful block in the current study, the reason was seen to be that the nerves where the block occurred later were the radial and ulnar nerves. It could be considered that the use of the double injection technique or administering a greater volume to these two nerves could prevent this situation.

Ferraro et al. used 1.56 ml of 1:200,000 adrenaline and bupivacaine 0.5%, and no pain was reported by any patient in the first three hours [14]. In the current study, the time of requirement for the first analgesia was accepted as VAS > 3. These times were determined to be a mean of 5.8 hrs for 7.5 ml and 16.6 hrs for 21 ml. A correlation was shown between the decrease in dose and the shortening of the time to the requirement for the first analgesia. In comparison with other studies, it is thought that although no vasoconstrictor agent was used, as bupivacaine is a long-acting agent, this could have prolonged the analgesia duration [9, 14-15].

## Conclusions

The application of an axillary block under USG guidance with a total of 7.5 ml of bupivacaine 0.5% as 2.5 ml per nerve can be considered to be sufficient to be able to provide surgical anaesthesia. However, it has been observed that at this volume, the time to onset of the sensory and motor blocks is prolonged, the time to block recovery is shortened, and the time to the requirement for additional analgesia is also shortened. There is a need for further studies to show that sufficient surgical anaesthesia could be provided at lower doses with appropriate imaging methods.
